# Chitosan increases *Pinus pinaster* tolerance to the pinewood nematode (*Bursaphelenchus xylophilus*) by promoting plant antioxidative metabolism

**DOI:** 10.1038/s41598-021-83445-0

**Published:** 2021-02-12

**Authors:** Marta Nunes da Silva, Carla S. Santos, Ana Cruz, Adrián López-Villamor, Marta W. Vasconcelos

**Affiliations:** 1 Universidade Católica Portuguesa, CBQF - Centro de Biotecnologia e Química Fina – Laboratório Associado, Escola Superior de Biotecnologia, Rua de Diogo Botelho 1327, 4169-005 Porto, Portugal; 2 Misión Biológica de Galicia (MBG-CSIC), Carballeira 8, Salcedo, 36143 Pontevedra, Spain

**Keywords:** Biotechnology, Microbiology, Plant sciences, Environmental sciences

## Abstract

The pine wilt disease (PWD), for which no effective treatment is available at the moment, is a constant threat to *Pinus* spp. plantations worldwide, being responsible for significant economic and environmental losses every year. It has been demonstrated that elicitation with chitosan increases plant tolerance to the pinewood nematode (PWN) *Bursaphelenchus xylophilus*, the causal agent of the PWD, but the biochemical and genetic aspects underlying this response have not been explored. To understand the influence of chitosan in *Pinus pinaster* tolerance against PWN, a low-molecular-weight (327 kDa) chitosan was applied to mock- and PWN-inoculated plants. Nematode population, malondialdehyde (MDA), catalase, carotenoids, anthocyanins, phenolic compounds, lignin and gene expression related to oxidative stress (thioredoxin 1, *TRX*) and plant defence (defensin, *DEF*, and a-farnesene synthase, *AFS*), were analysed at 1, 7, 14, 21 and 28 days post-inoculation (dpi). At 28 dpi, PWN-infected plants elicited with chitosan showed a sixfold lower nematode population when compared to non-elicited plants. Higher levels of MDA, catalase, carotenoids, anthocyanins, phenolic compounds, and lignin were detected in chitosan-elicited plants following infection. The expression levels of *DEF* gene were higher in elicited plants, while *TRX* and *AFS* expression was lower, possibly due to the disease containment-effect of chitosan. Combined, we conclude that chitosan induces pine defences against PWD via modulation of metabolic and transcriptomic mechanisms related with plant antioxidant system.

## Introduction

*Bursaphelenchus xylophilus*, commonly known as the pinewood nematode (PWN), is the etiological agent of the pine wilt disease (PWD), which affects several *Pinus* spp., particularly the maritime pine *P. pinaster*^1^. The PWD leads to important economic losses in the timber and wood industries, and increases the costs of disease management procedures and control, along with their inherent environmental impacts^2–4^. Currently, large areas of forests damaged by PWD are located in Japan, South Korea, China, and southern Europe, and these are estimated to increase by 50% in the next 50 years^5^.

Following infection, nematodes move and feed on plant resin canals leading to loss of water conductivity in stems and, subsequently, decreased transpiration and photosynthesis in leaves, resulting in generalized oxidative damage^6,7^. The production of hydrogen peroxide (H_2_O_2_) and superoxide ion radical (O^2·−^), for example, has been associated with increased *B. xylophilus* pathogenicity^8^. To counteract the harmful effects of these molecules, plants activate several antioxidant enzymes, such as superoxide dismutase, peroxidases and catalase. However, if excessive oxidative stress occurs, plant cells may accumulate malondialdehyde (MDA), a secondary product of cell wall lipid peroxidation^9^, whose content was already demonstrated to increase in plants inoculated with PWN^1^. In addition to the production of these metabolites, soluble phenolic compounds and lignin synthesis have also been implicated in plant defence against pathogens^10,11^. Phenolic compounds production and accumulation are associated with the browning of the leaf tissues injured by the PWN^12–14^, but the role of these secondary metabolites is still not fully understood. In addition, lignin biosynthesis was found to occur in stem tissues during the advanced stages of the PWD, and constitutive lignin was suggested to be a mechanical barrier against nematode invasion, conferring higher plant tolerance to the pathogens^15,16^. These enzymatic and other biochemical responses occur as soon as a few hours after infection, and are activated when a group of genes related to plant resistance recognizes pathogen effectors, initiating a resistance response^17^. In fact, genes related to secondary metabolite biosynthesis (α-farnesene synthase, *AFS*), defence against pathogens (defensins, *DEF*) and oxidative stress (thioredoxin, *TRX*) were found to be highly expressed in pine trees within a few hours after PWN infection^18^, especially when infected with virulent strains of *B. xylophilus*^16^.

Prevention is undoubtedly the best approach to reduce PWD incidence and different strategies have been suggested to avoid or treat the disease by: targeting the nematode itself, the vector, the host, or a combination of all three. Several synthetic compounds have already been developed to control the PWD, but many of them are toxic to the environment, labour intensive to apply, and expensive^19^. Previous studies suggested that chitosan can be used to enhance plant defence against bacteria^20,21^, fungi^22^ and nematodes^23,24^. Chitosan acts as a plant growth promoter, stimulating responses associated with both primary and secondary metabolism, including: carbon and nitrogen metabolism, primary photochemistry and photosynthesis, the tricarboxylic acid cycle, and terpenoid and phenolic compounds biosynthesis^25^. For example, carotenoids, one of the most important plant terpenoids, were found to be increased in basil and strawberry tissues following chitosan-elicitation^26,27^. Phenolic compounds, which play very important functions during pathogen infection (by e.g., providing mechanical strength through cell wall lignification processes) and in preventing oxidative stress, were also demonstrated to increase following elicitation with chitosan^28^. In fact, total phenolic content, and anthocyanins in particular, have been found to accumulate with chitosan treatment in leaf tissues of lemon balm and basil, and in cell cultures of grape vines^29–31^. Chitosan application also enhances the accumulation of several enzymatic antioxidants, including catalase, decreasing malondialdehyde (MDA) levels in leaf tissues thus improving plant antioxidant status^30,32^.

The use of chitosan in plant protection against pests and pathogens presents various advantages compared with the currently employed control compounds, as it is physically and biologically functional, biodegradable and biocompatible with tissues and cells^33,34^. Khalil et al.^23^ described the nematicidal activity of chitosan with different molecular weights (MW) against *Meloidogyne incognita* in tomato seedlings, reporting that low MW had the highest efficacy in controlling pathogen progression. Previous studies on the effect of chitosan as a control agent against the PWN demonstrated that low MW components had the highest nematicidal effect, reducing nematode density inside *P. pinaster* tissues up to 24 days post-inoculation (dpi)^24^. More recently, it has been demonstrated that *P. pinaster* and *P. pinea* supplemented with diazotrophic bacteria and a chitosan-producing fungus, *Cunninghamella elegans*, showed a 36-fold reduction of nematode colonization when compared to non-supplemented plants, as well as improved photosynthetic pigments, water content and phenolic compounds biosynthesis^35^. Despite the promising pieces of evidence that chitosan may be a useful tool in the control of the PWD, the physiological, metabolic and genetic mechanisms induced by chitosan application to PWN-infected *Pinus* spp. have not been addressed yet. The lack of knowledge regarding the regulatory mechanisms involved in the crosstalk between *Pinus* spp. plants and PWN after plant elicitation with chitosan greatly hinders the efforts to develop evidence-based, sustainable and affordable control methods against the PWD.

As such, in the present work, we hypothesized that the protective action of chitosan in PWN-infected plants might be due to systemic acquired resistance-related responses associated with a readjustment of the plant’s oxidative status. To test this hypothesis, the potential of low MW chitosan against the PWD in one-year-old *P. pinaster* plants was analysed, through the evaluation of the effect of chitosan on (1) nematode population dynamics, (2) plant antioxidative system and secondary metabolites accumulation, and (3) gene expression related to oxidative stress and defence responses.

## Results

### Nematode population in infected plants

**Figure 1:**
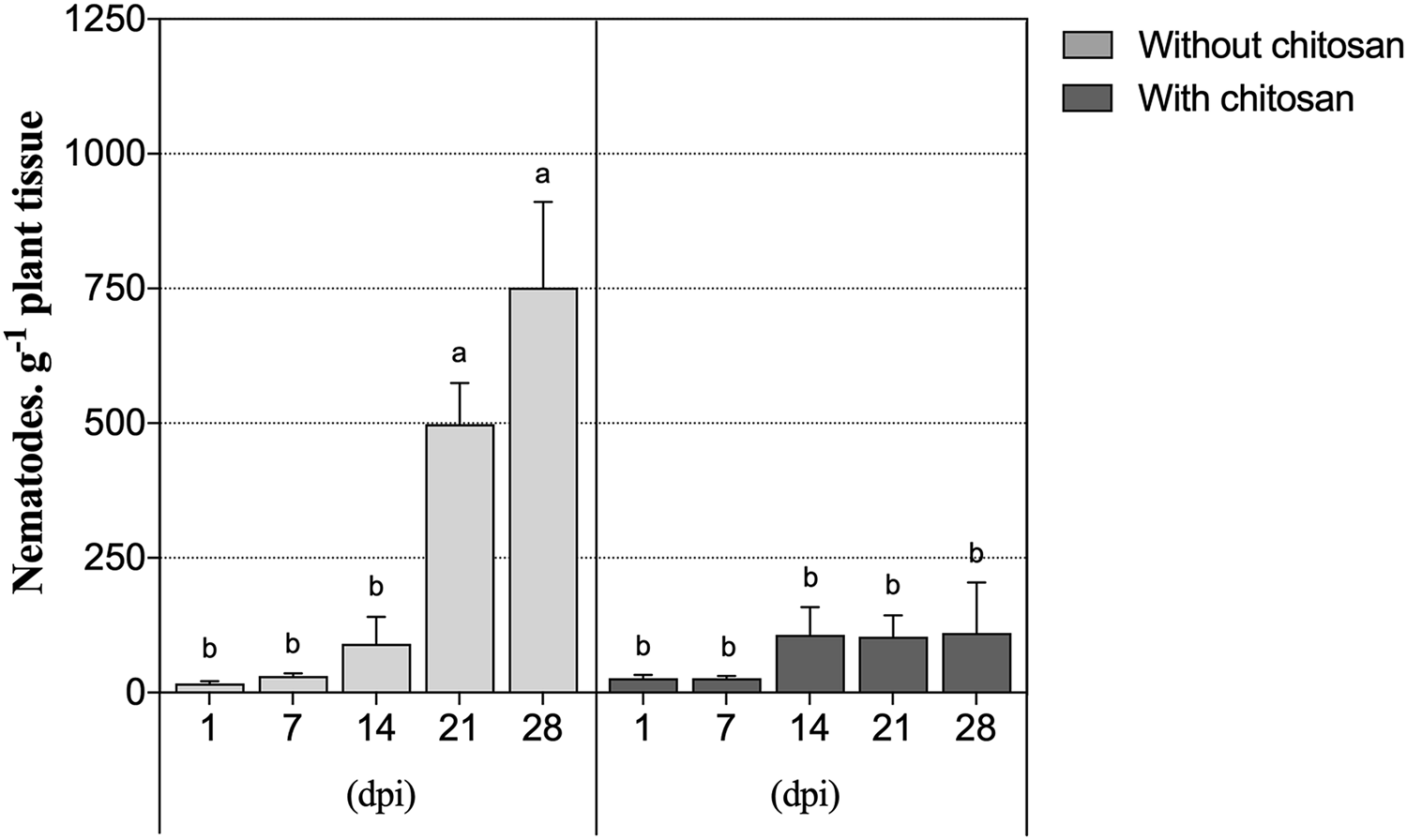
Nematode population in plant tissues. Nematode density in *P. pinaster* stem tissues 1, 7, 14, 21 and 28 days post-inoculation (dpi). Plants were inoculated with the pinewood nematode (PWN) *B. xylophilus* 14 days following the application of 0.5% acetic acid (without chitosan, non-elicited controls) or a chitosan solution to plants’ substrate. Data represents the difference between the averages of PWN-inoculated and water-inoculated plants ± SE, and different letters indicate statistically different means at *P* < 0.05.

Total nematode count inside stem tissues of inoculated plants without chitosan elicitation significantly increased by 44-fold from 1 to 28 dpi, indicating a successful infection and multiplication of PWN inside plant tissues over time (Fig. 1). In contrast, with chitosan elicitation the number of nematodes was low and statistically identical over the entire experimental period. In fact, only about 0.14-fold of the number of nematodes found in non-elicited plants were recovered from plants inoculated in the presence of chitosan, being the effect of the chitosan treatment significant (*P* < 0.0001) (Table 1).

**Table 1:** Effect of chitosan treatment, time-points of analysis (days post-inoculation, DPI) and their interaction in the physiological, biochemical and gene expression analyses of control and inoculated *P. pinaster* plants, following elicitation with chitosan.

	Chitosan treatment		DPI		Interaction	
Nematode population	F_(1,32)_	P value	F_(4,32)_	P value	F_(4,32)_	P value
	32.60	< 0.0001	19.75	< 0.0001	13.42	< 0.0001
MDA	F_(1, 26)_	P value	F_(4, 26)_	P value	F_(4, 26)_	P value
	13.94	0.0009	14.89	< 0.0001	0.7780	0.5496
Catalase activity	F_(1, 40)_	P value	F_(4, 40)_	P value	F_(4, 40)_	P value
	37.18	< 0.0001	34.58	< 0.0001	12.16	< 0.0001
Carotenoids	F_(1, 35)_	P value	F_(4, 35)_	P value	F_(4, 35)_	P value
	0.013	0.9116	13.02	< 0.0001	2.906	0.0355
Anthocyanins	F_(1, 30)_	P value	F_(4, 30)_	P value	F_(4, 30)_	P value
	0.324	0.5737	25.21	< 0.0001	4.488	0.0058
Total Polyphenols	F_(1, 33)_	P value	F_(4, 33)_	P value	F_(4, 33)_	P value
	19.24	0.0001	14.25	< 0.0001	1.219	0.3215
Lignin	F_(1,31)_	P value	F_(4, 31)_	P value	F_(4, 31)_	P value
	7.172	0.012	11.45	< 0.0001	2.415	0.0699
Gene expression	F_(1,20)_	P value	F_(4, 20)_	P value	F_(4, 20)_	P value
*TRX1*	34.37	< 0.0001	72.75	< 0.0001	42.92	< 0.0001
*DEF*	7.522	0.0125	10.83	< 0.0001	15.53	< 0.0001
*AFS*	10.10	0.0047	29.26	< 0.0001	20.77	< 0.0001

### Oxidative stress-related mechanisms

**Figure 2:**
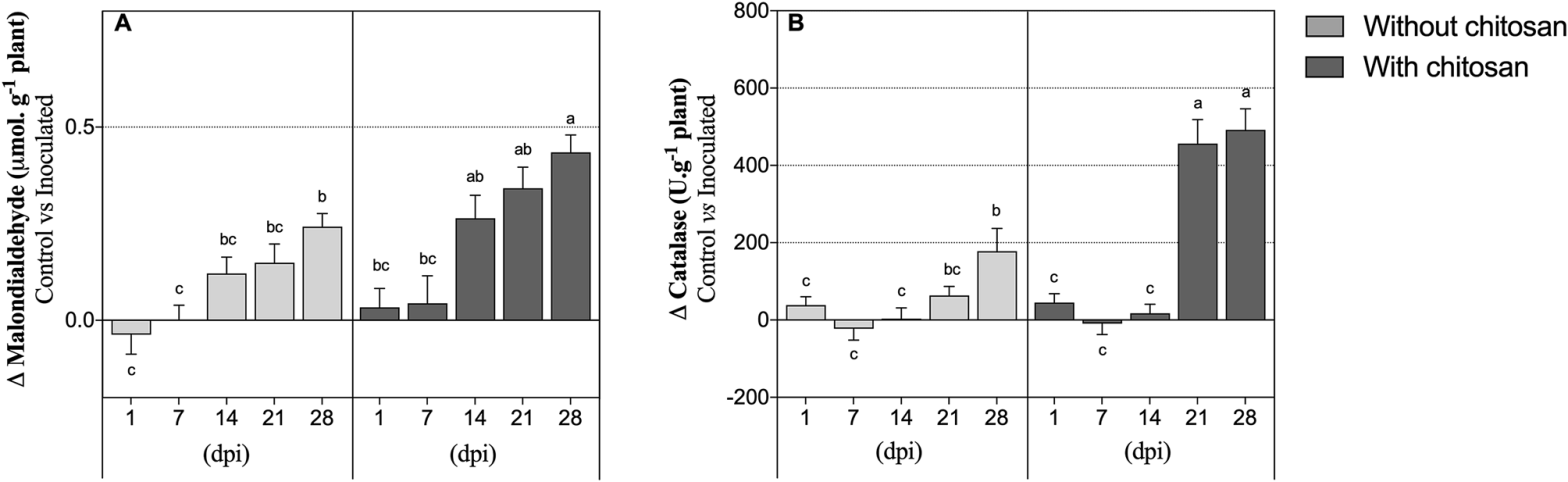
Lipid peroxidation and antioxidant activity. Malondialdehyde concentration and catalase activity in *P. pinaster* leaf tissues 1, 7, 14, 21 and 28 days post-inoculation (dpi) with the pinewood nematode (PWN) *B. xylophilus*. Plants were inoculated 14 days following the application of 0.5% acetic acid (without chitosan, non-elicited controls) or a chitosan solution to plants’ substrate. Data represents the difference between the averages of PWN-inoculated and water-inoculated plants ± SE, and different letters indicate statistically different means at *P* < 0.05.

Plants inoculated with PWN displayed a progressive and significant increase in MDA concentration throughout the experimental period, regardless of chitosan elicitation (Fig. 2A), with a significant effect of the chitosan treatment and the DPI (Table 1). In fact, at the end of the experimental period, PWN-inoculated plants elicited with chitosan had significantly higher MDA concentration than non-elicited plants (by 1.8-fold). Similarly to MDA, during the first 14 days of infection, catalase activity was not significantly affected in PWN-infected plants, regardless of chitosan elicitation, which remained similar to catalase levels in water-inoculated plants (Fig. 2B). Non-elicited inoculated plants presented a progressive increase in catalase levels and, at 28 dpi, these were 4.5-fold significantly higher than at 1 dpi. In chitosan-elicited plants, a more drastic and significant increase was registered after 14 dpi, with catalase activity increasing by 25-fold at 21 dpi, resulting in a significant interaction between chitosan treatment and DPI (Table 1).

**Figure 3:**
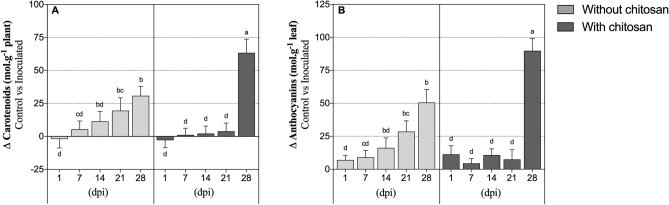
Carotenoids and anthocyanins. Carotenoids and anthocyanins concentrations in *P. pinaster* leaf tissues 1, 7, 14, 21 and 28 days post-inoculation (dpi) with the pinewood nematode (PWN) *B. xylophilus*. Plants were inoculated 14 days following the application of 0.5% acetic acid (without chitosan, non-elicited controls) or a chitosan solution to plants’ substrate. Data represents the difference between the averages of PWN-inoculated and water-inoculated plants ± SE, and different letters indicate statistically different means at *P* < 0.05.

Plants behaved similarly regarding the accumulation of carotenoids (Fig. 3A) and anthocyanins (Fig. 3B). From 1 to 28 dpi PWN-infected plants without chitosan elicitation showed a progressive and significant increase of 15- and sevenfold in carotenoid and anthocyanin concentrations, respectively. In chitosan-elicited plants, carotenoid and anthocyanin concentrations did not vary significantly between control and PWN-infected plants. Hence, no significant effect was found for the chitosan treatment for both analytes (Table 1). However, their concentration abruptly increased at 28 dpi by 21-fold in carotenoids concentration and 13-fold in anthocyanins concentration (when compared to the 21 dpi time-point), with a significant effect of the DPI and its interaction with the chitosan treatment (Table 1).

**Figure 4:**
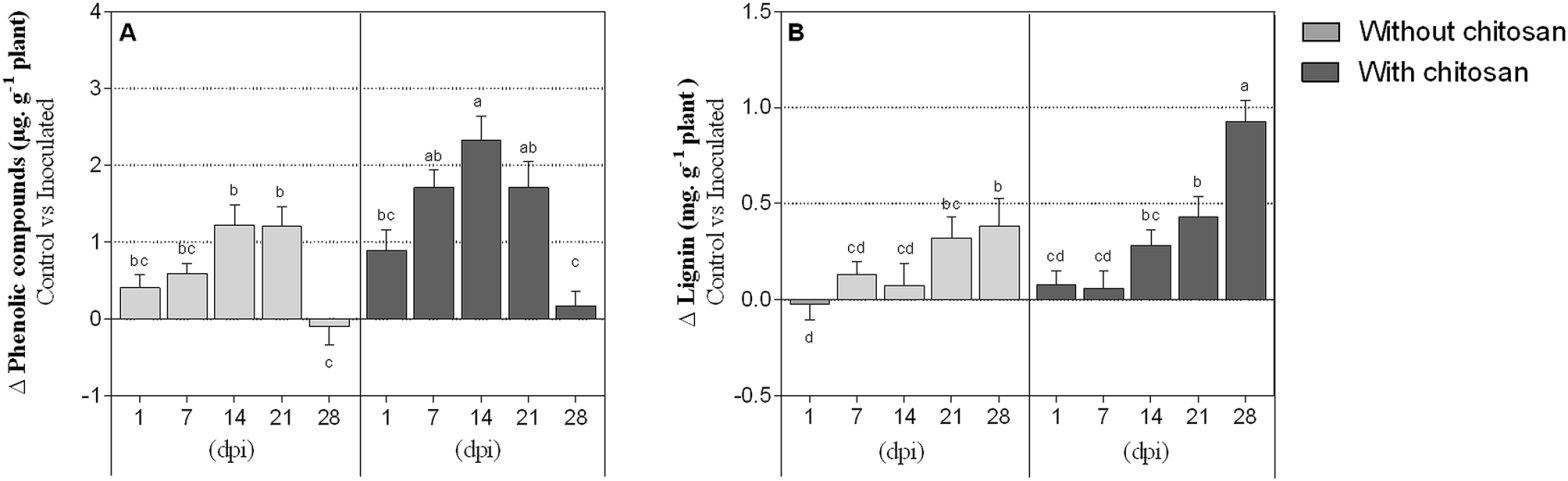
Total phenolic compounds and lignin. Total phenolic compounds and lignin concentrations in *P. pinaster* stem tissues 1, 7, 14, 21 and 28 days post-inoculation (dpi) with the pinewood nematode (PWN) *B. xylophilus*. Plants were inoculated 14 days following the application of 0.5% acetic acid (without chitosan, non-elicited controls) or a chitosan solution to plants’ substrate. Data represents the difference between the averages of PWN-inoculated and water-inoculated plants ± SE, and different letters indicate statistically different means at *P* < 0.05.

For all time-points, total phenolic concentration was about two times higher in PWN-infected plants with chitosan elicitation (*P* < 0.0001), when compared to non-elicited plants (Fig. 4A, Table 1). At 14 dpi, plants treated with chitosan presented the highest levels of total phenolic compounds. Moreover, in both elicited and non-elicited plants, polyphenols concentration was drastically reduced in infected plants at the end of the assay (28 dpi), returning to concentration levels similar to non-infected plants. In general, lignin concentration was similar between PWN-infected plants regardless of chitosan elicitation (Fig. 4B). However, at 28 dpi, inoculated plants showed increased lignin concentration (as compared with water-inoculated control plants), particularly with chitosan elicitation (by ca. twofold). No significant interaction between chitosan treatment and DPI was found for both total phenolic compounds and lignin accumulation (Table 1).

### Relative expression of stress-related genes

**Figure 5:**
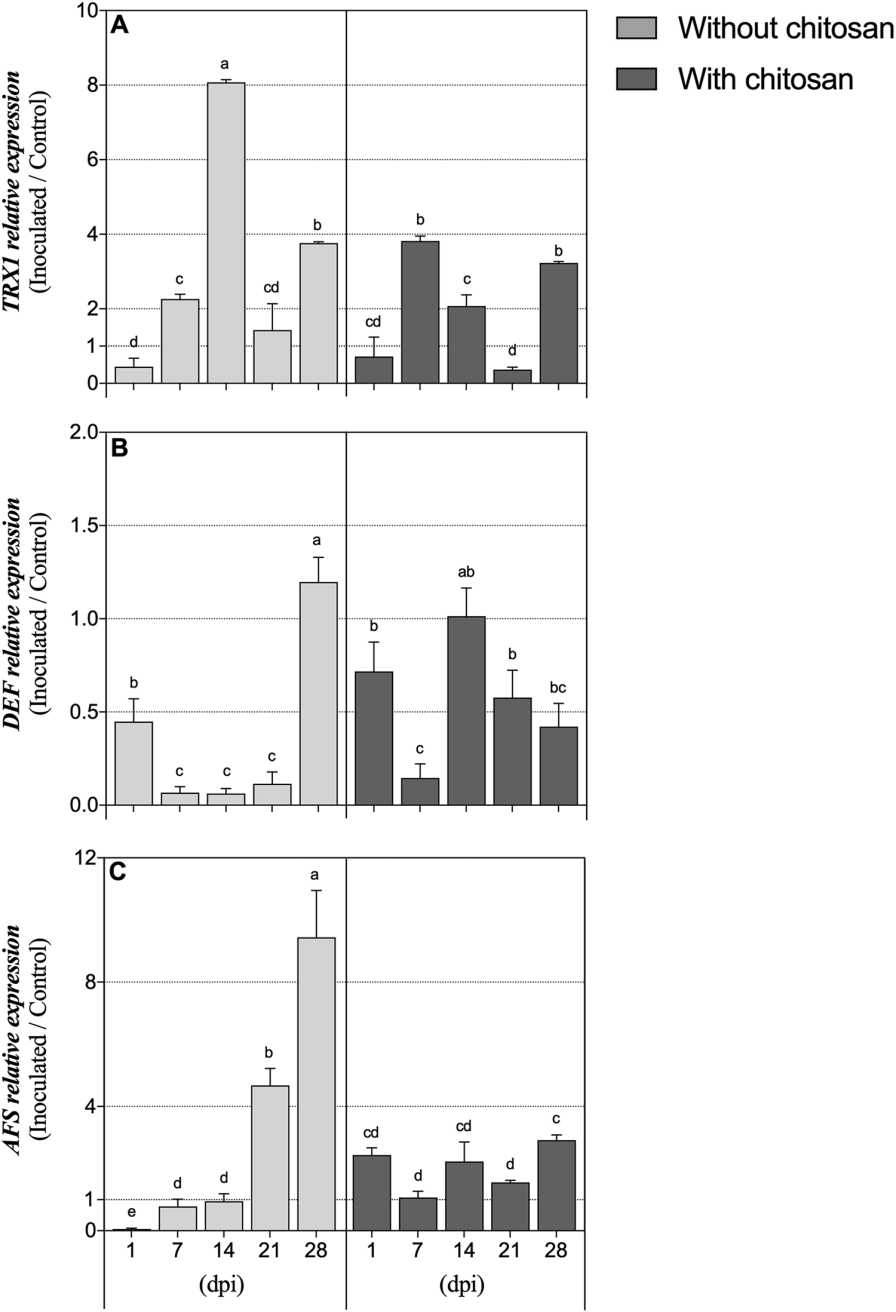
Stress-related gene expression. Fold expression of genes *thioredoxin H1(TRX1)* (**A**), *defensin (DEF)* (**B**) and *α-farnesene synthase (AFS)* (**C**) in plants inoculated with *B. xylophilus* relative to their expression in water-inoculated plants, 1, 7, 14, 21 and 28 days post-inoculation (dpi). Plants were inoculated 14 days following the application of 0.5% acetic acid (without chitosan, non-elicited controls) or a chitosan solution to plants’ substrate. Data represents the difference between the averages of PWN-inoculated and water-inoculated plants ± SE, and different letters indicate statistically different means at *P* < 0.05.

Regarding the expression of *TRX1* gene (Fig. 5A), non-elicited PWN-infected plants showed an increase by more than twofold at 7 dpi, which was followed by an eightfold increase at 14 dpi, when compared to 1 dpi. The expression decreased to less than half of the values seen at 14 dpi, and remained at this level until the end of the experiment. For elicited PWN-infected plants, *TRX1* relative expression was also significantly increased at 7 dpi, however, it immediately decreased in the following time-points and was generally lower than the expression values in non-elicited plants. Following PWN infection, *DEF* expression (Fig. 5B) was differently modulated throughout the experiment depending on the chitosan treatment (*P* < 0.05). In non-elicited plants, from 1 to 7 dpi it was significantly reduced to almost null values and maintained at this level until 21 dpi, until its expression increased again, by about twofold, at 28 dpi. In elicited plants, there was a significant decrease in *DEF* expression at 7 dpi, but at 14 dpi its expression increased again, remaining unaltered until the end of the experimental period. Chitosan elicitation significantly impacted the expression pattern of *AFS* (Fig. 5C), with non-elicited PWN-infected plants having a progressive increase in its expression over time, reaching the highest value at 28 dpi (ninefold higher than at 1 dpi). Elicited plants showed constant and relatively low *AFS* expression throughout the experiment, which was twofold higher in PWN-infected plants than in controls. Chitosan treatment and DPI had a significant effect in the expression of all genes, also having a significant interaction among them (Table 1).

## Discussion

When the PWN infects susceptible plant species, there is a primary phase of the disease (early phase), where the nematode population remains low, without producing any visual symptoms. When the second phase develops (advanced stage), PWN population increases exponentially, beginning a more destructive phase for the plant due to nematode feeding and reproduction on the epithelial cells^6,7,36^. Previous histological and biochemical works on the PWN population dynamics demonstrated that the PWD progresses strongly between 7 and 14 days after infection^37,38^, which is in accordance to what was observed in the present work in the absence of chitosan elicitation. It has been previously shown that chitosan application prevents nematode multiplication in plant tissues over time^24^, and reduces the number of juveniles in soil^39^. The present work seems to attest for the promising role of chitosan elicitation of *P. pinaster* plants against the PWD, as the nematode population remained at low levels throughout the entire experimental period, indicating that this compound prevented, or, at least, slowed disease progression.

In susceptible *Pinus* spp. host plants, PWN damages xylem and phloem parenchyma and cortex cells, which can compromise water transport, lead to the peroxidation of the unsaturated lipids of cellular membranes and to ROS formation, culminating in leaf necrosis and often in plant death^7,40^. MDA, a secondary compound of lipid peroxidation reactions, is frequently used as an indicator of cell damage induced by free radicals^41^. Interestingly, despite preventing nematode reproduction in plants tissues, chitosan did not prevent cellular damage, leading to increased MDA accumulation. This could be a result of the abrupt induction of plant defence mechanisms upon PWN infection, with consequent formation of ROS^42^. To counteract the negative effects of intracellular ROS levels, increased or de novo synthesis of several antioxidant enzymes occur. One of those enzymes, catalase, can capture H_2_O_2_ and convert it in water and oxygen molecules, which do not have a damaging impact to plant cells^43,44^. Here, following PWN inoculation, catalase activity was significantly induced throughout the progression of the disease, although more drastically in chitosan-elicited plants, putatively representing the activation of plants’ antioxidant defences. Hydrogen peroxide-mediated oxidative burst has been observed in several plant species supplemented with chitosan, and it is generally acknowledged that it is required for chitosan-induced defence responses^25,45^. Increased H_2_O_2_ content in chitosan-elicited plants in generally accompanied by the accumulation of several antioxidant enzymes, among which catalase and peroxidase seem to be the most important^46–48^. The application of chitosan may, therefore, lead to a stronger, and possibly more efficient, antioxidant response against PWN infection, which may contribute to an increased tolerance against the pathogen.

Carotenoids and anthocyanins are metabolites involved in plant protection by preventing photosystem damage, participating in signalling, ROS scavenging and inhibiting the growth of noxious microorganisms^49–51^. It appears that their concentration increased in a similar manner to the PWN population, MDA concentration and catalase activity, as they gradually increased in inoculated plants (as compared to the controls), in both elicited and non-elicited plants. Previous studies have reported that chitosan-elicitation promotes plant defences through increased activity of several defence-related enzymes, e.g. phenylalanine ammonia-lyase, involved in the biosynthesis of phenolic compounds and terpenoids (which include, among several others, carotenoids and anthocyanins)^52,53^.We hypothesise that increased concentrations of carotenoids and anthocyanins in PWN-infected tissues, especially by the end of the experimental period, may be a coping mechanism to prevent further cellular damage caused by the pathogen^54^. As observed for catalase, chitosan elicitation seems to promote the accumulation of these metabolites, thus corroborating their potential in improving plants’ antioxidant defences. Along with carotenoids and anthocyanins biosynthesis, phenolics and lignin are also involved in plant resistance to biotic stress^15,55,56^. In general, total phenolic compounds concentration increased at 14 dpi (with statistical significance in PWN-infected elicited plants), which seems to correspond to the beginning of the advanced stage of the PWD. This is concordant with a previously reported positive relationship found between nematode migration and phenolics concentration^57^. In the case of lignin, chitosan-elicited plants had higher accumulation than non-elicited ones, which may indicate an intensified tissue lignification after pathogen infection, possibly increasing plant tolerance to the pathogen^58^. Chitosan also induces a generalized H_2_O_2_-mediated hypersensitive response, involved in cell wall lignification processes that confer protection against pathogen-induced mechanical damage^30,59^. The higher accumulation of carotenoids, anthocyanins, phenolics and lignin observed in the present study, particularly during the later stages of PWN infection and with chitosan elicitation, are in agreement in these previous works, and attest the potential of chitosan elicitation in promoting *Pinus* spp. defences against the pathogen.

In plants, thioredoxins act as signalling molecules through their reduction-regulatory properties in response to pathogen attacks^60,61^. In the present work, significant overexpression of *TRX1* was observed in non-elicited plants at 14 dpi, corroborating the hypothesis that the advanced stage of the PWD was triggered around this time-point. In PWN-infected elicited plants, *TRX1* expression levels were not induced at this time-point, which is coherent with the lower nematode population and increased antioxidative activity previously discussed. Plant defensins, on the other hand, are usually associated with antimicrobial and antifungal activity, constituting the first defensive line against pathogens^62,63^. It is interesting to note that, here, *DEF* expression pattern was very distinct between chitosan-elicited and non-elicited plants following PWN infection. It seems that, without chitosan elicitation, plants were not able to activate the expression of this gene as the PWN population progressed, whereas in elicited plants *DEF* expression was significantly increased along time, possibly contributing to the lower extent of disease progression observed. Also involved in plant defence is α-farnesene (*AFS*), an herbivore-induced plant volatile, usually increased in plants suffering pathogen attacks, being key in disease resistance^18,64^. Here, non-elicited plants which showed increased nematode population over time, also displayed higher levels of *AFS* expression, particularly at 28 dpi, when the highest number of nematodes were registered. Contrastingly, chitosan-elicited plants had lower levels of *AFS* expression, possibly due to the low nematode progression and multiplication within their tissues.

In general, chitosan increased *P. pinaster* tolerance to the PWN, resulting in decreased nematode density, increased accumulation of metabolites involved in antioxidant activity, and differential expression of stress-related gene expression. This further supports the potential of chitosan in the prevention and/or treatment of the PWD in *Pinus* spp., which should be further explored and considered for the development of sustainable disease control strategies.

## Methods

### Plant maintenance and chitosan elicitation

One-year-old (40–50 cm height) *P. pinaster* plants were maintained in a growth chamber (Fitoclima 10 000 EHF; Aralab, Rio de Mouro, Portugal) under a 16 h light/8 h darkness photoperiod at 25/18 °C, respectively, and 80% relative humidity. Photon flux density during the day was 380 µmol m^−2^ s^−1^. Plants were kept in a commercial substrate (COMPO SANA universal substrate; Compo GmbH, Münster, Germany) composed of (mg L^-1^): 200–450 N; 200–500 P_2_O_5_; 300–550 K_2_O, pH 5.0–6.5. Plants were watered weekly until field capacity.

An acidic solution (0.5% of acetic acid, pH 6.0) of 4.4% chitosan (molecular weight 327 kDa, deacetylation degree ≥ 75%, Ref.: 448869, Sigma-Aldrich, Missouri, USA) was prepared and left shaking for 24 h before use. A single chitosan treatment was applied to a group of 75 plants by adding 80 mL the chitosan solution to the substrate of each plant. An additional group of 75 plants served as non-elicited control, where a 0.5% solution of acetic acid (no chitosan) was added to the soil.

### Nematode culture and plant inoculation

Fourteen days following plant elicitation, a virulent strain of PWN (65 GO, isolated from Góis, Portugal) was inoculated into plant stems. Nematodes were maintained in agar plates with *Botrytis cinerea* mycelia for 14 days, after which they were extracted from the growing media using the Baermann funnel technic^65^. Plant inoculation was performed with 2 000 nematodes at *ca*. 3 cm from the top of each plant as described by Nunes da Silva et al.^24^.

### Experimental design and plant sampling

A total of 150 *P. pinaster* plants were used in this experiment. Chitosan was applied to a group of 75 plants, in which 25 were mock-inoculated with water (non-inoculated controls) and 50 were inoculated with PWN. An additional group of 75 plants served as non-elicited control, where a 0.5% solution of acetic acid (without chitosan) was added to the substrate: 25 plants were mock-inoculated with water (non-inoculated controls) and 50 were inoculated with PWN. At 1, 7, 14, 21 and 28 days post-inoculation (dpi) five biological replicates of each treatment were sampled. At each time-point, leaves were recovered for the quantification of lipid peroxidation (MDA), catalase, carotenoids and anthocyanins, and stems were used for total soluble phenolics and lignin quantification and gene expression analysis. Five additional PWN-inoculated plants were sampled at the same time-points for whole-stem PWN quantification.

### Nematode quantification in plant tissues

In each time-point, five inoculated plants from each treatment were randomly selected for PWN quantification. Leaves were removed, and the entire stem was cut into small portions (*ca*. 0.5 cm) and used for nematode quantification using the Baermann funnel technique for 24 h at 25 °C. Nematode density in stem tissues was estimated taking into consideration whole stem fresh weight.

### Lipid peroxidation and catalase

Quantification of MDA, a sub-product of lipid peroxidation, was performed by mixing vigorously 100 mg of leaf tissue previously homogenized in liquid nitrogen with 10 mL of 0.5% thiobarbituric acid in 20% of trichloroacetic acid and incubated at 100 °C for 30 min. After the incubation period, the reaction was terminated by transferring the tubes into ice. Samples were centrifuged during 10 min at 5 000 g and the supernatant was filtered, after which absorbances were measured at 450, 532 and 600 nm and MDA estimated as described by Yang et al.^66^.

Quantification of catalase activity was performed following the protocol by Ruley et al.^67^, adding 1.5 mL of phosphate buffer 1 M to 100 mg of leaf tissue previously homogenized with liquid nitrogen. Samples were vigorously mixed for 2 min, centrifuged at 5 000 g for 10 min at 4 °C and the supernatant was recovered and diluted 3 times with phosphate buffer. Three hundred and forty-four microlitres of a 73 mM H_2_O_2_ solution (in 0.5 M Tris/HCl pH 7.0) were then added to 666 μL of sample extract. Enzyme activity was measured for 3 min at 240 nm. One unit of enzyme corresponded to a decrease of 0.001 in absorbance.

### Plant secondary metabolites

For carotenoids and anthocyanins quantification, leaves sampled as previously described (100 mg) were homogenized with liquid nitrogen and extracted with 10 mL of cold acetone/Tris buffer solution at 1 M (80:20 v/v, pH 7.8). Samples were incubated at 4 °C for 72 h and absorbances were recorded at 470, 537, 647 and 663 nm. Metabolite concentration was calculated as described by Sims and Gamon^68^.

Total soluble phenolic compounds were quantified as in Azevedo et al.^69^. Lyophilized leaf tissues previously homogenized with liquid nitrogen (100 mg) were extracted with 5 mL of methanol for 24 h in the dark at 4 °C. One hundred μL of sample extract were mixed with 5 mL of ultrapure water and 0.5 mL of Folin-Denis reagent, stirred vigorously and incubated at room temperature for 5 min. After incubation, 1.5 mL of sodium carbonate at 35% (w/v) was mixed with each sample and let to react in the dark for 2 h, after which 2.9 mL of ultrapure water were added and absorbances were determined at 760 nm. Soluble phenolic compounds were determined using a quercetin calibration curve.

Lignin was determined by the acetyl bromide method^70^ using the leaf biomass used for phenolics extraction, which was subjected to sequential 24 h extractions with water, acetone and hexane. Samples were dried at 60 °C for 48 h, and 10 mg of biomass were mixed with 500 µL of glacial acetic acid and 500 µL of acetyl bromide 25%. The mixture was digested at 50 °C for 2 h with vigorous stirring, after which samples were centrifuged for 10 min at 15 000 g. One hundred microlitre of sample were recovered to a new microcentrifuge tube and homogenized with 200 µL of glacial acetic acid, 150 µL of NaOH 0.3 M, 50 µL of hydroxylamine hydrochloride 0.5 M and 500 µL of glacial acetic. Absorbances were recorded at 280 nm and lignin concentration in each sample was determined through a standard calibration curve.

### Gene expression analysis

From the five biological replicates from each treatment, three were randomly selected for gene expression analysis. After stem tissue homogenization with liquid nitrogen, RNA extraction was performed using the RNeasy Mini Kit (QIAGEN GmbH, Hilden, Germany) following the manufacturer’s protocol. RNA quality and quantity were determined spectrophotometrically and single-strand cDNA was synthesised using iScrip cDNA Synthesis Kit (Bio-Rad Laboratories, Inc, California, USA) following the manufacturer’s instructions. The transcript levels of genes encoding proteins related to oxidative stress (thioredoxin 1, *TRX*) and plant defence (defensin, *DEF*; α-farnesene synthase, *AFS*) were analysed. Primer sequences were retrieved from available published literature^18^. Reactions of RT-qPCR were carried out in a thermal cycler CFX96 Touch Deep Well Real-Time PCR Detection System using iQ SYBR Green Supermix (Bio-Rad Laboratories, Inc, California, USA) and data visualized with the software Bio-Rad CFX Manager 3.1. The amplification protocol cycle was 95 °C for 3 min and 40 cycles at 95 °C for 15 s, 30 s at 54 °C and 71 °C for 30 s. The comparative CT method (ΔΔCT) was used for the relative quantification of gene expression values using the geometric mean of the expression of the control transcripts ubiquitin and 18S ribosomal RNA genes, and the plants inoculated with water (non-inoculated controls) as reference sample^71^. For each sample and target gene, two technical replicates were analysed.

### Statistical analysis

Data were analysed using GraphPad Prism v6.0 software (GraphPad Software, California, USA) and significant differences between chitosan treatments were determined using two-way analysis of variance (ANOVA) followed by Tukey’s post hoc test with *P* = 0.05. Due to the large number of datasets, and to facilitate result viewing and discussion, results concerning MDA, catalase, carotenoids, anthocyanins, phenolic compounds and lignin are presented as the difference between the averages of PWN- and water-inoculated plants. Bars showing positive values represent an increase in PWN-inoculated plants relatively to water-inoculated plants, whereas negative results represent a decrease. Propagation of uncertainty was used to calculate the standard error of each value.
